# Evaluation of a New Entomopathogenic Strain of *Beauveria bassiana* and a New Field Delivery Method against *Solenopsis invicta*

**DOI:** 10.1371/journal.pone.0158325

**Published:** 2016-06-24

**Authors:** Jun Li, Qiang Guo, Miaofeng Lin, Lu Jiang, Jingwen Ye, Dasong Chen, Zhigang Li, Jianqing Dai, Shichou Han

**Affiliations:** 1 Guangdong Key Laboratory of IPM in Agriculture and Public Laboratory of Wild Animal Conservation and Utilization, Guangdong Institute of Applied Biological Resources, Guangzhou, Guangdong Province, P.R. China; 2 Shenzhen Wild Animal and Plant Protection Administration Agent, Shenzhen, China; Northwest A&F University, CHINA

## Abstract

*Solenopsis invicta* Buren is one of the most important pests in China, and control measures are mainly based on the use of synthetic pesticides, which may be inadequate and unsustainable. Hence, there is a growing interest in developing biological control alternatives for managing *S*. *invicta*, such as the use of entomopathogenic fungi. To facilitate the commercialization of entomopathogenic fungi against S. invicta, 10 Beauveria bassiana isolates originating from different hosts were tested for virulence in laboratory bioassays, and the most pathogenic strain, ZGNKY-5, was tested in field studies using an improved pathogen delivery system. The cumulative mortality rate reached 93.40% at 1×10^8^ mL^-1^ conidia after 504 h. The germination and invasion of the spores were observed under a scanning electron microscope, and several conidia adhered to the cuticle of *S*. *invicta* after 2 h. Furthermore, the germ tubes of the conidia oriented toward the cuticle after 48 h, and the mycelium colonized the entire body after 96 h. Based on the efficacy observed in the laboratory trials, further experiments were performed with ZGNKY-5 strain to evaluate its utility in an injection control technology against *S*. *invicta* in the field. We found that three dosage treatments of ZGNKY-5 strain (500 mL, 750 mL, and 1,000 mL per nest) had significant control effects. Our results show that this strain of *Beauveria bassiana* and our control method were effective against *S*. *invicta* in both laboratory and field settings.

## Introduction

*Solenopsis invicta* Buren (Hymenoptera: Formicidae) was originally distributed in the Parana River basin of South America (Paraguay and Parana rivers) [1], including western Brazil, Argentina, Paraguay, and the Panama Canal [2–4]. In mainland China, *S*. *invicta* was first detected in September 2004 in Wuchuan, Guangdong Province [5]. *S*. *invicta* can sting repeatedly and will vigorously attack anything that disturbs their mound. Some people have allergic reactions to *S*. *invicta* stings that range from rashes and swelling to paralysis, or anaphylactic shock. *S*. *invicta* also build mounds that interfere with the maintenance of lawns, park lands, recreational turf areas, roadsides, and agricultural lands. They can be found foraging for food or even nesting inside homes and are known to invade electrical boxes and destroy wiring. In addition, they can actually cause plant damage by direct feeding or by protecting and encouraging certain plant sucking insects which cause plant damage. The Chinese government is currently exploring optimal control methods. In China, *S*. *invicta* is mainly distributed in city wharfs, schools, public green spaces, fitness facilities, and parks [6], because these locations are near water resources. However, because these locations are also frequented by people, the use of synthetic pesticides for prevention and control of *S*. *invicta* carries great risks. For example, the use of synthetic pesticides can result in pesticide residues and high levels of insecticide resistance, which are costly and undesirable [7–11]. This practice not only pollutes the environment and affects water quality, but is also harmful to humans and animals. Thus, there is a growing interest in microbial biocontrol agents such as entomopathogenic fungi for use in pest management programs. Unlike synthetic pesticides, entomopathogenic fungi have little effect on non-target insects, therefore, they have high selectivity, as well as high efficiency, and they are inexpensive to produce, generate no pollution, and are innocuous to humans and animals.

*Beauveria bassiana* (Bals.) Vuill. (Hypocreales: Cordycipitaceae) is an entomopathogenic fungus that is widely studied and used worldwide, it can infect 15 orders, 149 families, and 700 species of insects [12]. Several studies have demonstrated the effects of *B*. *bassiana* on target insect pests [13]. Zimmermann [14] reported that the relative safety and specificity of this microbial biocontrol agent facilitated its acceptance by growers for use it in pest management programs. Thus, entomopathogenic fungi have been successfully developed worldwide as control agents for several agricultural pests. Certain species are already commercially available as biological insecticides for the control of various pests [15, 16]. The efficiency of *B*. *bassiana* at various concentrations and using various application methods for controlling colonies of *S*. *invicta* have been studied in laboratory and field settings [17, 18]. Siebeneicher et al. [19] suggested that *B*. *bassiana* is one of the most promising natural enemies of *S*. *invicta* for its biological control. *B*. *bassiana* had a high pathogenicity against *S*. *invicta* [20]. Oi et al. [18] found that *B*. *bassiana* can reduce the foraging opportunities of these ants and can improve the competitiveness of other ant species. Bextine and Thorvilson [21] found that the use of baits with *B*. *bassiana* can reduce the activity of *S*. *invicta*. Pereira [22] studied the spread of *B*. *bassiana* in nests of *S*. *invicta* and the relationship between *B*. *bassiana* and the nest soil. Recent research in China has tested several *B*. *bassiana* strains that are highly virulent against *S*. *invicta* [23–26] and have considerable potential as biological control agents. Unfortunately, there has been little commercialization of entomopathogenic fungi as biological control agents for *S*. *invicta*.

In this paper, we report the screening of 10 new strains of the entomopathogenic fungus *B*. *bassiana*, that originated from different hosts against *S*. *invicta* in the laboratory. Strains with the highest pathogenicity were evaluated further, and the germination and invasion of spores were observed using Scanning Electron Microscopy. Based on the efficacy results from the laboratory trials, the best isolate was evaluated in the field against *S*. *invicta* nests with a new field application method that we designed.

## Materials and Methods

### Ethics Statement

No specific permissions were required for these locations/activities.

None of the species used in this study are endangered or protected.

### Insect colony

Several colonies of *S*. *invicta* were obtained from Panyu, Guangzhou, China, and the species was confirmed with a morphological examination. The ants were reared in 35×16×9 cm plastic boxes that contained soil under laboratory conditions of 25±1°C, 70±5% RH and an 8:16 L:D photoperiod at the Guangdong Institute of Applied Biological Resources (GIABR). The inner walls of the boxes were coated with Fluon ^**®**^(AGC Chemicals Europe, Ltd., Thornton-Cleveleys, Lancashire, UK) to prevent the ants from escaping. All the colonies were composed of numerous workers, a functional queen or queens, and brood in various stages of development. Each box with ants was provided with *Corcyra cephalonica* (Stainton), *Tenebrio molitor*, honey solution (honey: water = 1:3, V:V), and sterile water. The honey solution and sterile water were placed in the 15 mL glass tubes covered with cotton. The ants were separated from the soil by dripping water two days before the experiment and were reared in 28×17×18 cm plastic boxes that contained a 1 cm thickness 2CaSO_4_·H_2_O on the bottom to dry the ants. To extract the ant colony from the soil, water was slowly dripped into the plastic boxes, with constant observation the water level. The ants (workers, queen(s), winged males and females) and worker ants carrying brood (eggs, larvae, and pupae) will move to the top of the soil and ultimately float or “raft” on the surface of the water. Using a slotted spoon, the ants and brood were scooped out and placed in a plastic box [27]. Again, the inner walls of the boxes were coated with Fluon, and the feeding method was the same as described above.

### *B*. *bassiana* source

The origin and source of the 10 fungal isolates are shown in S1 Table. The maintenance of isolates and the production of conidia were conducted on potato dextrose agar (PDA) at 25±1°C under continuous darkness. The conidial concentrations were determined with a hemocytometer and adjusted with sterile water and Tween-80 at 0.05% (v/v) [13]. The viability of the conidia was confirmed on a PDA medium [28], and it was > 90% for all strains.

### Screening of the 10 new fungal isolates

The effect of the fungal isolates on the survival of *S*. *invicta* was evaluated by treating *S*. *invicta* with 1×10^8^ mL^-1^ conidia, which is the commonly used concentration in spray applications for the control of *S*. *invicta* in China [23–26]. Sterile water containing Tween-80 at 0.05% (v/v) was used in the control treatment. *S*. *invicta* were dipped for 5 s in the conidial suspension. All the *S*. *invicta* were transferred to 15×10×7 cm plastic boxes with filter paper on the bottom to dry the ants. The inner walls of the boxes were coated with Fluon. Each box with ants was provided honey solution (honey: water = 1:1, V:V) and sterile water, which was placed in a 5mL glass tube covered with cotton. The plastic boxes were stored in a climatic chamber (25±1°C, RH 70±5% and a 2:22 L:D photoperiod). The number of dead *S*. *invicta* was recorded at various time points (2, 12, 24, 36, 48, 60, 72, 84, and 96 h). The corrected mortality of *S*. *invicta* was used as an indication of mycosis. Each replicate consisted of approximately 500 ants, and each treatment was replicated three times. Because ZGNKY-5 strain showed the greatest mortality of *S*. *invicta*, the number of dead *S*. *invicta* treated with the strain was additionally recorded at the following time points (120, 144, 168, 192, 240, 336, 384, 456, and 504 h).

### Scanning electron microscope (SEM) observations

For the SEM observation, *S*. *invicta* treated with the strain ZGNKY-5 (2, 12, 24, 36, 48, 60, 72, 84, and 96 h) were fixed in 2.5% glutaraldehyde for 12 h, then, they were flushed by 0.1 mol/L phoric acid buffer (pH = 7.4) 3 times, 10 min each time. Next, the ants were dehydrated in 70% ethanol for 2 h and kept dehydrated in an ascending series of ethanol (75, 80, 85, 90, 95 and 100%, 6 min each)[29]. They were left to air dry for a few seconds and mounted on SEM stubs with double-sided carbon tape. The dried samples were sputtered with gold and observed in a SEM under Quanta 200 FEG at high-vacuum mode.

### Field control experiments with different dosages of the ZGNKY-5 strain against *S*. *invicta*

The ZGNKY-5 strain was identified as the most virulent strain for the ants in the previous laboratory experiment and was selected for further evaluation in a field experiment. The ants used in this experiment were confirmed to be *S*. *invicta* by morphological examination. The experiments were carried out in several selected nests (approximately 20×30×10 cm each) in Panyu, Guangzhou, beginning on April 15, 2015. The mean temperature and humidity during this period were 30°C (25–33°C) and 60% (40–80%). The mean temperature and humidity at 5 cm under the mound were 28°C (23–30°C) and 65% (45–85%). Worker ants from each nest were trapped using a 20 mL trapping bottle containing a small piece of sausage, and the number of ants was counted before each the treatments. Then, five doses of ZGNKY-5 strain (100 mL, 250 mL, 500 mL, 750 mL, and 1,000 mL per nest) were applied, with a control control treatment sterile water containing Tween-80 at 0.05% (v/v). Each replicate consisted of 5 nests, and every treatment was replicated three times. In addition, we designed a new type of injector for microbial control of *S*. *invicta*, which was used in this experiment as well. The injector consists of a Syringe Injection Head S1 Fig, a body, and a bottle filled with the suspensions of fungal conidia (1×10^8^ mL^-1^). In order to control the ants in different depth of the nests in the field, the Syringe Injection Head consists of 3 retractable steel tube with spray hole on each tube, and each tube is approximately 20 cm long, of which diameter is 0.8cm, 1cm, 1.2cm respectively S1 Fig. The conidia of strain were injected into the nests in the field with the injector for the control of *S*. *invicta* S2A Fig. The control solution and suspensions were prepared as described above. The worker ants were trapped as described above on days 4, 10, 20 and 30 after the treatment, and the numbers of ants were counted. Each nest was covered with a transparent plastic cylinder (diameter = 30 cm, height = 20 cm) without top and bottom cover S3 Fig, in which the worker ants were trapped, the outer walls of the plastic cylinder were coated with Fluon to prevent ants from other nests from being trapped. The trapping bottle with bait was laid beside the mound and inside the plastic cylinder. Then, the worker ants were trapped for approximately 0.5–1.0 h and were counted. Meanwhile, each mound was poked with a stick, then, the activity nests was recorded with which more than 3 live ants emerged within 60 s[30]. The numbers of active nests and worker ants trapped from each treatment and control were used to calculate the decreasing rate of nests and worker ants, which was used as an indication of the treatment effect. The calculation formula is as follows:

$$\text{Decreasing rate of nests}=\left[\text{1}-\frac{M_{\text{0}}\times TM_{\text{1}}}{TM_{\text{0}}\times M_{\text{1}}}\right]\times 100\%$$ (1)

*M*_0_: the number of active nests in the control before the treatment; *TM*_0_: the number of active nests in treatment area before the treatment; *TM*_1_: the number of active nests in the treatment area after the treatment; *M*_1_: the number of active nests in the control after the treatment.

$$\text{Decreasing rate of worker ants}=\left[\text{1}-\frac{W_{\text{0}}\times TW_{\text{1}}}{TW_{\text{0}}\times W_{\text{1}}}\right]\times 100\%$$ (2)

*W*_0_: the number of worker ants trapped by trapping bottle in control before the treatment; *TW*_0_: the number of worker ants trapped by trapping bottle in the treatment area before the treatment; *TW*_1_: the number of worker ants trapped by trapping bottle in the treatment area after the treatment; *W*_1_: the number of worker ants trapped by trapping bottle in the control after the treatment.

### Data analysis

All the experiments were conducted in three replications. Mortality data were corrected with the control mortality using the Abbott’s formula in the screening experiment [31]. Percentage data were arcsine square-root transformed before statistical analysis was performed using SPSS 16.0 software (SPSS Inc. Chicago, IL, USA, 2004). Oneway analysis of variance (ANOVA) was utilized to analyze differences among treatments. Differences among varied treatments then were determined using the Tukey’s multiple comparison, all p-values using Tukey’s Test adjustments to control for type I error. In all experiments, differences among means were considered significant at P = 0.05. For the field trials, the decreasing rate of nests and worker ants were compared over all sample dates by ANOVA, using P<0.05 to determine significance.

## Results

### Screening of the 10 new fungal isolates

After 10 days of treatments with the 10 new strains of *B*. *bassiana* (1×10^8^ conidia mL^-1^) in the laboratory, corrected mortality of *S*. *invicta* treated by 5 strains (QB3.428, QB3.45, ZGNKY-1, ZGNKY-3, and ZGNKY-5) showed a significant difference from the control, and strain ZGNKY-5 was the most efficient, with 80.26% mortality S4 Fig. Hence, strain ZGNKY-5 was the most active against *S*. *invicta* S4 Fig and significantly reduced the worker survival (F = 34.57; df = 10, 22; P<0.001; S4 Fig).

In addition, S5 Fig shows that strain ZGNKY-5 was more effective than the control ant killing *S*. *invicta*. The cumulative mortality difference was not obvious at 72 h before the treatments, but it was noticeable after 96 h. In particular, the cumulative mortality rate reached more than 80% and 93.40% after 240 and 504 h, respectively (F = 132.47; df = 12. 26, P < 0.001; S5 Fig).

### Scanning electron microscope observations (SEM)

As shown in S6 Fig, when the workers were treated with 1×10^8^ conidia mL^-1^ of *B*. *bassiana* strain ZGNKY-5, several conidia adhered to the cuticle of *S*. *invicta* after 2 h S6A Fig. The germ tubes of the conidia oriented toward the cuticle after 48 h S6B Fig. The mycelium emerged from the antenna of *S*. *invicta* after 72 h. Finally, the mycelium colonized the entire body after 96 h S6D Fig.

### Field control experiments with different dosages of strain ZGNKY-5 against *S*. *invicta*

Nests of *S*. *invicta* were treated for 30 days with the five dosages of strain ZGNKY-5 (100 mL, 250 mL, 500 mL, 750 mL, and 1,000 mL per nest). Decreasing rate of nests of the two lowest dosages (100 mL and 250 mL per nest) showed no difference from the control at day 4 (F = 19.75; df = 5,12; P<0.001; S7A Fig. However, the three higher dosages (500 mL, 750 mL, and 1,000 mL per nest) each showed a significant difference from the control at day 10 (F = 35.98; df = 5,12; P<0.001; S7B Fig, at day 20 (F = 123.10; df = 5,12; P<0.001; S7C Fig, and at day 30 (F = 73.72; df = 1,5; P<0.001; S7D Fig).

Compared with the control, all treatments showed a significant reduction in the number of worker ants. However, the decreasing rate of worker ants of the three highest doses (500 mL, 750 mL, and 1,000 mL per nest) showed a more significant difference from the control than other did the other dosages (100 mL and 250 mL per nest) with control at day 4 (F = 95.16; df = 5,12; P<0.001; S8A Fig), at day 10 (F = 58.38; df = 5,12; P<0.001; S8B Fig), at day 20 (F = 670.77; df = 5,12; P<0.001; S8C Fig), and at day 30 (F = 294.35; df = 1,5; P<0.001; S8D Fig).

## Discussion

Entomopathogenic fungi are used for the control of several Lepidoptera and Coleopteran pests of grains and horticultural crops in the fields and greenhouses [16, 32]. Several reports refer to the study of entomopathogenic fungi against *S*. *invicta*. For example, after treatment with *B*. *bassiana* (1×10^8^ conidia mL^-1^) in the laboratory, Yang et al. [24] found that the cumulative mortality of *S*. *invicta* was greater than 70% after 10 days. Moreover, 80% of *S*. *invicta* died because they were infected with an isolate of *B*. *bassiana* obtained from *S*. *invicta* in Brazil [20, 33]. Lv [25] found that the Bb02 strain isolated from *Ostrinia nubilalis* and the Bb04 strain isolated from *Bactrocera dorsalis* had high pathogenicity against *S*. *invicta*. Hence, *B*. *bassiana* has a high insecticidal activity against *S*. *invicta*.

In this study, after screening 10 new strains of *B*. *bassiana* against *S*. *invicta* in the laboratory, our results demonstrate that strain ZGNKY-5 was the most active isolate against *S*. *invicta*. To verify that *B*. *bassiana* germinated on the body surface of *S*. *invicta*, penetrated its cuticle, and eventually led to the insect’s death, spores were observed under SEM, after the strains with high pathogenicity were screened out. According to our SEM observations, the conidia of *B*. *bassiana* strain ZGNKY-5 penetrated the *S*. *invicta* cuticle soon after germination. Our results differ slightly from those of Vestergaard et al. [34] and Wang et al. [35]. In their studies, several fungi germ lines produced appressoria within 24–48 h post-inoculation on *F*. *occidentalis*. Wang et al. [36] infected *Plutella xylostella* (Linnaeus) with *Metarhizium anisopliae*. The conidia germinated after 10 h and penetrated the larva cuticle of *P*. *xylostella* after 13 h. You et al. [37] observed that the spores of *B*. *bassiana* infected the larvae of *P*. *xylostella* when they germinated at 12 h, and the conidia penetration occurred in any part of *P*. *xylostella* after 36 h. In this study, the conidia of the ZGNKY-5 strain were observed germinating and penetrating on the cuticle of *S*. *invicta* individuals, which, eventually, leading to their death. However, the conidia of the ZGNKY-5 strain on *S*. *invicta* required more time to penetrate the ant cuticle than those of the other insects mentioned above. This may because *S*. *invicta* has a harder cuticle [38], making it difficult for the conidia of the ZGNKY-5 strain to penetrate after germination. The longer time required for penetration might also be due to the differences among species of entomopathogenic fungi or hosts.

In recent years, research on the use of fungi to control *S*. *invicta* has been increasing. However, most of the studies have been performed in the laboratory, with only a few evaluating the potential of entomopathogenic fungi as field biological control agents for *S*. *invicta*. The main reason may be the lack of suitable application methods. In particular, because of the biological characteristics of *S*. *invicta*, which live underground, it is difficult to use entomopathogenic fungi to control *S*. *invicta* in the field, Moreover, although entomopathogenic fungi have several advantages in the control of many pests, such as being highly selective, highly efficient, inexpensive to produce, non-polluting, and innocuous to humans and animals, certain limitations remain in the application of entomopathogenic fungi in the field. UV light, low relative humidity and high temperatures can cause the applied conidia to have a relatively short persistence [39]. For example, the conidia of *M*. *anisopliae* and *B*. *bassiana* persisted for only 1–2 days on cowpea leaves after the application [40]. Likewise, Inglis et al. [41] reported similar results with *B*. *bassiana* on alfalfa foliage. For these reasons, in our field experiment, we designed a new tool and new methods. The conidia of strain ZGNKY-5 were injected into the nests in the field with an injection control technology (designed in-house) against *S*. *invicta*. We found that most nests did not show any activity after 30 days S2B Fig, and there were large numbers of dead ants near the nests S2C Fig. After a nest was treated, it was excavated and inspected after 30 days, and we observed that the dead bodies of *S*. *invicta* were covered with fungus S2D Fig. Finally, the dead insects were collected and brought to the laboratory. We performed tests to confirm that strain ZGNKY-5 was the pathogenic agent. The field experiment further supported the laboratory efficacy of strain ZGNKY-5, which resulted in a consistent decrease in the numbers of nests and worker ants of *S*. *invicta*. Therefore, the results of this research show that the strain ZGNKY-5 produced a high mortality against *S*. *invicta* both in the laboratory and in the field. Because the conidia were injected inside the nests, we may have avoided the limitations caused by the effects of UV light, low relative humidity, and high temperature. Furthermore, injecting the conidia inside the nests may have provided favorable conditions for a increased effectiveness of the fungus. The results show that *B*. *bassiana* has excellent potential for application in the biological control of *S*. *invicta*. This outcome is consistent with the conclusion of Siebeneicher [19].

Based on these observations, we suggest the use of this new strain, ZGNKY-5, of *B*. *bassiana* and our new injection control technology against *S*. *invicta* in the field. Our data support the use of *B*. *bassiana* as a biological control agent against *S*. *invicta*. Indeed, this method of control significantly reduced the survival of *S*. *invicta* in the laboratory and the field.

## Supporting Information

S1 Fig Syringe injection: head of the injector designed by our team for the microbial control against *S*. *invicta*. (EPS)

S2 Fig An injection control technology (designed in-house) against *S*. *invicta* in the field. (A) The conidia of strain ZGNKY-5 were injected into the nests in the field; (B) The treatment after 30 days; (C) The dead ants near the nest; (D) The dead bodies of *S*. *invicta* were covered with fungus.
(TIF)

S3 Fig A transparent plastic cylinder without top and bottom covers prevented ants of other nests from being trapped. Note: The trapping bottle containing bait was laid beside the mound which is inside the plastic cylinder.
(EPS)

S4 Fig Corrected mortality of 10 isolates against *S*. *invicta* at ten days post-treatment in the laboratory. Note: Bars represent the standard error of the means (based on three independent replicates, each consisting of approximately 500 adults). All strains were tested at 1×10^8^ conidia mL^-1^.
(EPS)

S5 Fig Cumulative mortality of ants at different time points during the 504 h after treatment with the ZGNKY-5 strain in the laboratory. Note: Data are expressed as the means ± SEM based on three replicates, each consisting of approximately 500 ants. The ZGNKY-5 strain was applied at 1×10^8^ conidia mL^-1^. Treatments marked with different letters are significantly different from each other (P<0.05). The same conditions apply for the following figures.
(EPS)

S6 Fig Germination and infection of *B*. *bassiana* strain ZGNKY-5 conidia on the cuticle of *S*. *invicta*. (A) Conidia adhering to the cuticle of *S*. *invicta*; (B) Germ tube of conidia oriented toward the cuticle; (C) Mycelium emerging from the antenna of *S*. *invicta*. (D) The mycelium colonized the entire body of *S*. *invicta*.
(TIF)

S7 Fig Decrease rate of nest after treatment of *S*. *invicta* with various doses of strain ZGNKY-5. (A) The decrease rate of nest at day 4; (B) The decrease rate of nest at day 10; (C) The decrease rate of nest at day 20; (D) The decrease rate of nest at day 30.
(EPS)

S8 Fig Decrease rate of worker ants after treatment of *S*. *invicta* with various doses of strain ZGNKY-5. (A) The decreasing rate of worker ants at day 4; (B) The decreasing rate of worker ants at day 10; (C) The decreasing rate of worker ants at day 20; (D) The decreasing rate of worker ants at day 30.
(EPS)

S1 Table Origin of the *B*. *bassiana* fungal isolates screened against *S*. *invicta*. (DOC)
