# Supplementary figures and images for: Evaluation of a New Entomopathogenic Strain of Beauveria bassiana and a New Field Delivery Method against Solenopsis invicta
Source: PLoS One. 2016 Jun 24;11(6):e0158325. doi: 10.1371/journal.pone.0158325 (PMC4920426; doi:10.1371/journal.pone.0158325)

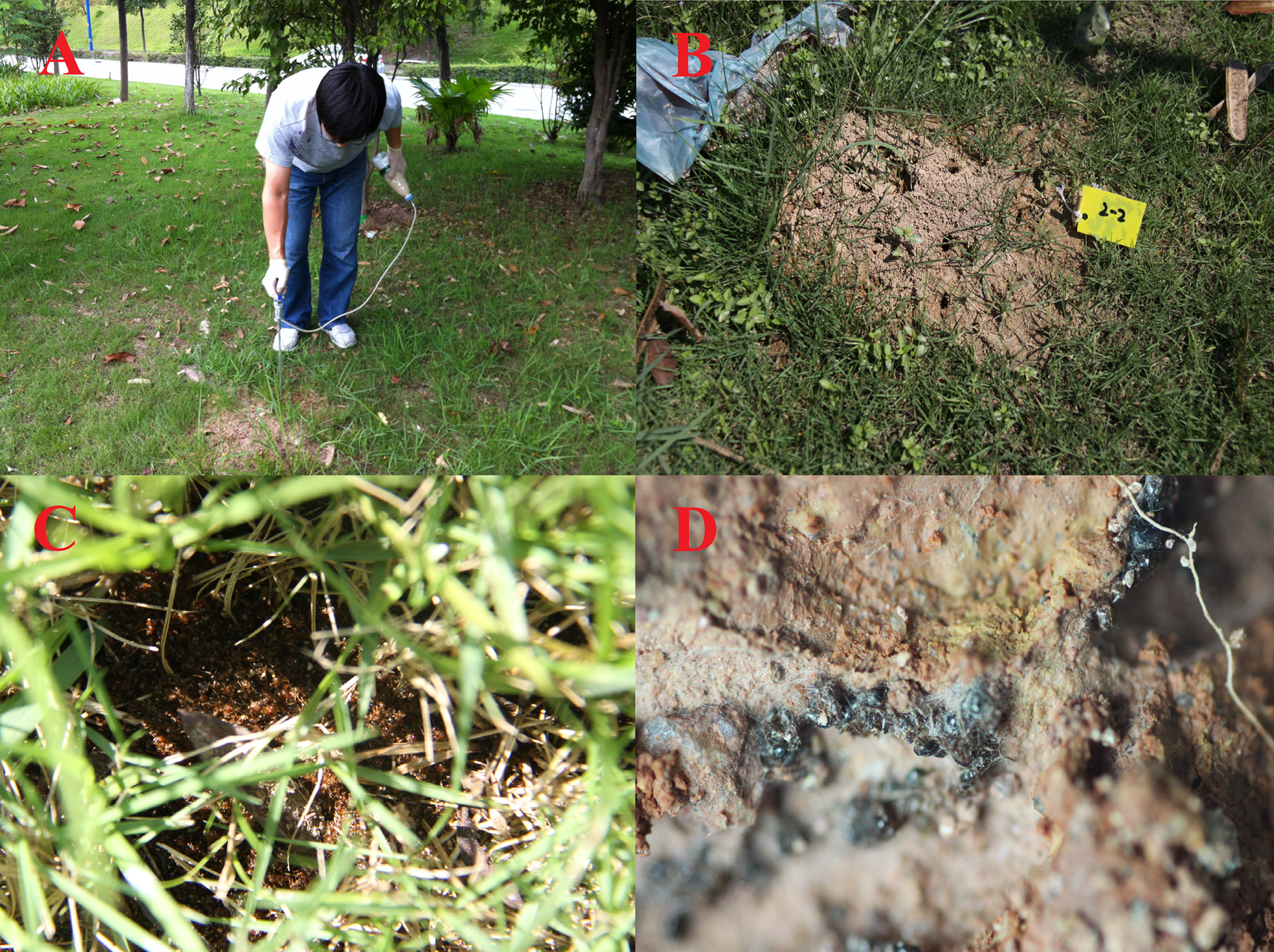

Supplement: S2 Fig — (A) The conidia of strain ZGNKY-5 were injected into the nests in the field; (B) The treatment after 30 days; (C) The dead ants near the nest; (D) The dead bodies of S. invicta were covered with fungus. (TIF) [file pone.0158325.s002.tif]

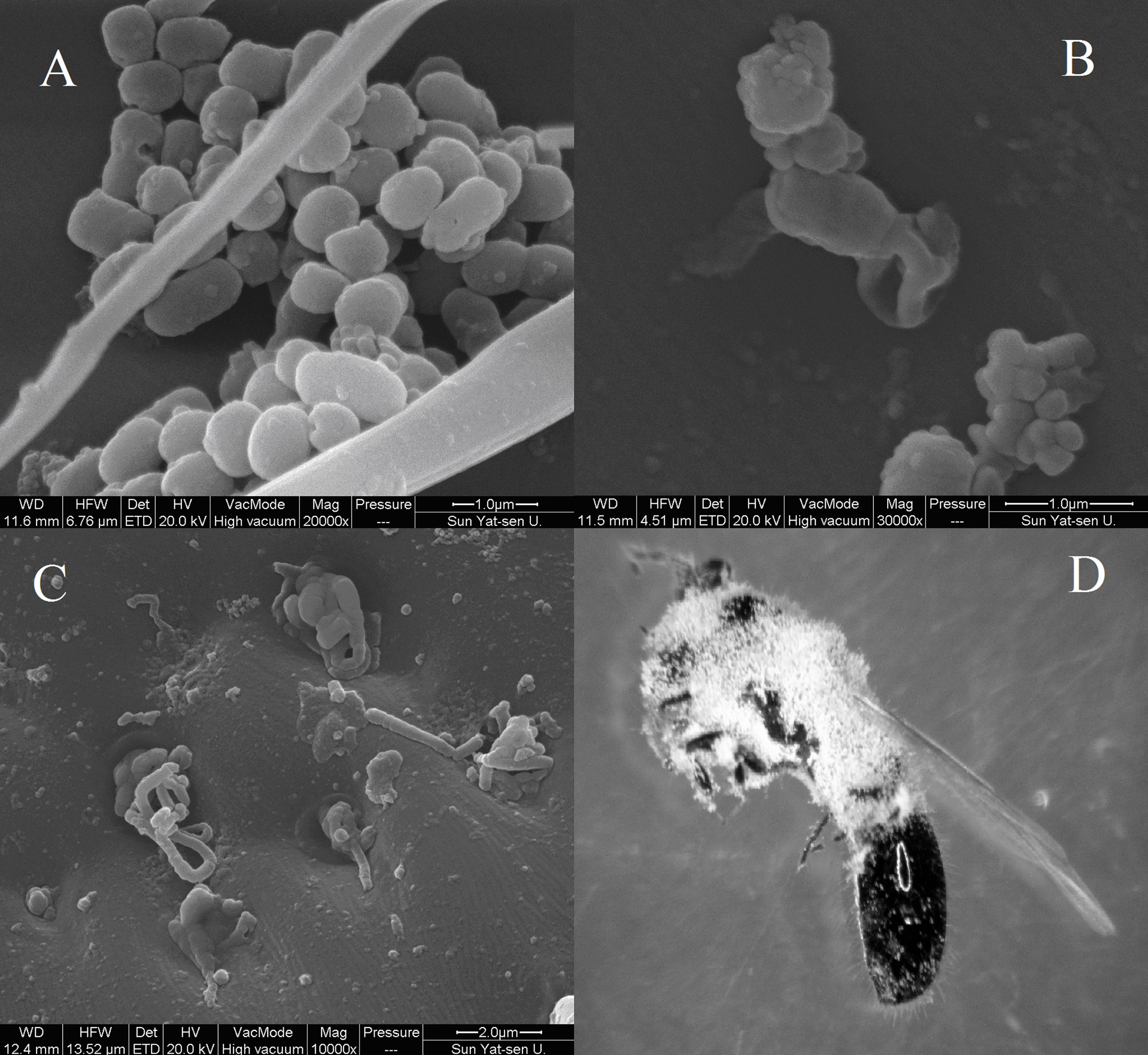

Supplement: S6 Fig — (A) Conidia adhering to the cuticle of S. invicta; (B) Germ tube of conidia oriented toward the cuticle; (C) Mycelium emerging from the antenna of S. invicta. (D) The mycelium colonized the entire body of S. invicta. (TIF) [file pone.0158325.s006.tif]
