# Supplementary material for: Evaluation of a New Entomopathogenic Strain of Beauveria bassiana and a New Field Delivery Method against Solenopsis invicta
Source: PLoS One. 2016 Jun 24;11(6):e0158325. doi: 10.1371/journal.pone.0158325 (PMC4920426; doi:10.1371/journal.pone.0158325)
Supplement: S1 Table — (DOC) [file pone.0158325.s009.doc]

Table 1. Origin of the *B. bassiana* fungal isolates screened against *S. invicta*.

| Fungal isolates | Host or source of origin | Site of origin (collection date) |
| --- | --- | --- |
| QB3.428 | *Ostrinia nubilalis* (Lepidoptera: Pyralidae) | Changchun, Jilin (2014) |
| QB3.45 | *Dendrolimus punctatus punctatus* Walker (Lepidoptera: Dendrolimus) | Guangzhou, Guangdong (2014) |
| QB3.46 | *Ostrinia nubilalis* (Lepidoptera: Pyralidae) | Guan, Hebei (2014) |
| QB3.436 | *Brown rice planthopper* (Nilaparvata lugens (Stal: Homoptera: Delphacidae) | Philippines (2013) |
| ZGNKY-01 | *Ostrinia nubilalis* (Lepidoptera: Pyralidae) | Wrumqi, Xinjiang (2015) |
| ZGNKY-1 | *Ostrinia nubilalis* (Lepidoptera: Pyralidae) | Beijing (2015) |
| ZGNKY-2 | *Ostrinia nubilalis* (Lepidoptera: Pyralidae) | Beijing (2015) |
| ZGNKY-3 | *Ostrinia nubilalis* (Lepidoptera: Pyralidae) | Beijing (2015) |
| ZGNKY-4 | *Ostrinia nubilalis* (Lepidoptera: Pyralidae) | Suining, Liaoning (2015) |
| ZGNKY-5 | *Ostrinia nubilalis* (Lepidoptera: Pyralidae) | Chaozhou, Guangdong (2015) |
